# Effect of the initial pH on the anaerobic digestion process of dairy cattle manure

**DOI:** 10.1186/s13568-022-01486-8

**Published:** 2022-12-28

**Authors:** Job Jonathan Castro-Ramos, Aida Solís-Oba, Myrna Solís-Oba, Carlos Ligne Calderón-Vázquez, Jesús Mireya Higuera-Rubio, Rigoberto Castro-Rivera

**Affiliations:** 1 Instituto Politécnico Nacional, Centro de Investigación en Biotecnologia Aplicada, 90700 Tepetitla de Lardizábal, Tlaxcala Mexico; 2 Universidad Autónoma Metropolitana, Unidad Xochimilco, Ciudad de Mexico, Mexico; 3 Instituto Politécnico Nacional, CIIDIR Unidad Sinaloa, 81100 Guasave, Sinaloa Mexico

**Keywords:** Volatile fatty acids, Indole-3-acetic acid, Gibberellic acid, Anaerobic digestion, Cow manure

## Abstract

Anaerobic digestion (AD) has recently been studied to obtain products of greater interest than biogas, such as volatile fatty acids (VFAs) and phytoregulators. The effect of the initial pH of cow manure and the fermentation time of the AD on the microbial composition, VFAs, indole-3-acetic acid (IAA) and gibberellic acid (GA_3_) production was evaluated. The cow manure (7% solids) was adjusted to initial pH values of 5.5, 6.5, 7.5, and 8.5, and the AD products were analyzed every four days until day 20. The initial pH and the fermentation time had an important effect on the production of metabolites. During AD, only the hydrolytic and acidogenic stages were identified, and the bacteria found were from the phyla *Firmicutes, Bacteroidetes, Actinobacteria,* and *Spirochaetes*. The most abundant genera produced in the four AD were *Caproiciproducens, Clostridium *sensu stricto* 1*, *Romboutsia, Paeniclostridium, Turicibacter, Peptostreptococcaceae, Ruminococcaceae* and *Fonticella*. The highest amount of VFAs was obtained at pH 8.5, and the production of the acids was butyric > acetic > propionic. The maximum production of GA_3_ and IAA was at an initial pH of 6.5 on day 20 and a pH of 5.5 on day 4, respectively. There was a strong correlation (> 0.8) between the most abundant microorganisms and the production of VFAs and GA_3_. The anaerobic digestion of cow manure is a good alternative for the production of VFAs, GA_3_ and IAA.

**Supplementary Information:**

The online version contains supplementary material available at 10.1186/s13568-022-01486-8.

## Introduction

The volume of manure generation around the world is enormous because of the increasing demand for meat/dairy products, with concomitant environmental contamination due to its inadequate disposal, but anaerobic digestion (AD) is an option for the treatment of manure (Li et al. 2021).

AD involves a sequence of chemical processes in which biodegradable organic waste is broken down by microorganisms in the absence of oxygen, which is useful for organic waste reduction, sludge stabilization, and biological toxicity reduction (Huang et al. 2019; Khatami et al. 2021). Different kinds of microorganisms participate in each of the four stages of AD: hydrolysis, acidogenesis, acetogenesis, and methanogenesis (Greses et al. 2020). These microorganisms live in obligate mutualistic cooperation, and the complete performance of AD is dependent on this complex syntrophic relationship. Changes in one group can alter the chain of anaerobic sequences and, consequently, the complete AD process (Nguyen et al. 2019).

Knowledge of the microbial population in each stage, as well as their metabolic properties and interactions, is necessary to improve the performance of AD. The microorganisms and the ratios of the metabolites produced in each stage of AD can change considerably in response to the organic loading rate, type and concentration of the substrate, temperature, and pH (Sträuber et al. 2012; Khatami et al. 2021). The metabolic pathways and microorganisms involved in the methanogenic stage have been extensively studied, whereas the preceding stages are less known (Sträuber et al. 2012). Probably the most recognized product from AD is biogas; however, other metabolites from the earlier stages are also highly important, with a great variety of applications, such as volatile fatty acids (VFAs) (Pervez et al. 2022; Khatami et al. 2021; Nzeteu et al. 2021) and phytohormones (Li et al. 2016).

VFAs are short-chain aliphatic monocarboxylate compounds containing two to six carbon atoms, such as acetic, propionic, butyric, isobutyric, valeric, isovaleric, and caproic acids. VFAs are important starting materials for the production of biofuels, olefins, alcohols, ketones and biodegradable plastics (Wang et al. 2019; Khatami et al. 2021; Greses et al. 2020), and are widely used in the textile, food, cosmetics, and pharmaceutical industries (Nzeteu et al. 2021). The current costs of butyric, acetic and propionic acids are 1500–1650, 400–400 and 2000–2500 euros/ton, respectively (Khatami et al. 2021). Currently, VFAs are produced from petroleum-derived compounds or by natural extraction methods; however, their production from more environmentally sustainable processes has attracted the attention of researchers. For example, AD is very attractive because it is cost-effective and is a way to recover value-added products from waste (Pervez et al. 2022; Khatami et al. 2021). During AD, VFAs production will be preferred if the growth of methanogens is inhibited. The substrate and methods used such as heat shock, pH increase/decrease or freeze–thaw treatments make the AD unsuitable for methanogens, with a preference for the hydrolytic and acidogenic stages (Pervez et al. 2022; Bermúdez-Penabad et al. 2017). To produce VFAs from AD, different types of organic waste have been explored, such as food (Peng et al. 2018; Zhou et al. 2018; Khatami et al. 2021), fruit (Li et al. 2022), tuna wastes (Bermúdez-Penabad et al. 2017), and sewage sludge from a wastewater treatment plant (Wang et al. 2019), and maize silage (Sträuber et al. 2012).

On the other hand, the plant hormones gibberellic acid (GA_3_) and indole-3-acetic acid (IAA) are used to stimulate the development of plants and improve the yields and quality of crops (Karishma et al. 2019). GA_3_ is usually produced in aerobic submerged fermentation systems by the aerobic fungus *Gibberella fujikuroi* and *Fusarium moniliforme* (Shukla et al. 2003; Oliveira et al. 2017) or by the bacteria *Methylobacterium oryzae* (Siddikee et al. 2010); however, the high cost of production, low GA_3_ yield, and high energy consumption limit extensive production of GA_3_ (Tian-Qiong et al. 2017). IAA is mainly produced by aerobic fermentation in axenic cultures using pure substrates (Tian-Qiong et al. 2017). Some works have shown that GA_3_ and IAA can also be produced during AD (Li et al. 2016); however, information regarding GA_3_ and IAA production under anaerobic conditions is limited.

Currently, research focused on the use of green technologies has increased steadily, the application of AD of organic waste is of particular interest, because valuable products are obtained from materials that can pollute the ecosystem and are difficult to dispose of. From the AD of cow manure were obtained important metabolites like IAA and GA_3_ useful in agriculture, and VFAs useful in different types of industries. The AD can be considered as a comprehensive solution to take advantage of organic residues and reduce the environmental pollution caused by them, and to produce useful metabolites from cheaper sources and eco-friendly production processes. Besides the importance of the initial pH and fermentation time during anaerobic digestion, it is important to characterize the microbial communities involved in the process, to determine their effect on the production of VFAs, IAA and GA_3_, and identify the conditions that maximize their production.

The aim of this work was to evaluate the effect of the initial pH and fermentation time during the anaerobic digestion of dairy cattle manure on the composition of the microbial communities involved in the fermentation process, and the production of VFAs, IAA and GA_3_.

## Materials and methods

### Anaerobic digestion

The cow manure (total solids 14.00% of fresh matter (FM), volatile solid 11.76% FM, pH 6.55) was collected from the dairy cattle module of the Instituto Tecnológico del Altiplano de Tlaxcala, México, and the animals were fed a standard forage diet. A composite sample of ten subsamples of fresh cow manure was made. The manure sample was mixed with distilled water to a final concentration of 7% total solids and used for the AD. The pH of the diluted cow manure was 6.5, and it was adjusted to 5.5, 7.5 or 8.5 by the addition of HCl (37%) or Na_2_CO_3_.

The anaerobic digestion was carried out in 120 mL serum bottles filled with 70 mL of the mixture of manure. Nitrogen was pumped in to displace the air in the headspace of each serum bottle and ensure anaerobic conditions. Then, the bottles were capped with rubber stoppers, sealed with a metal ring and incubated at 37 °C for 20 days. A completely random design with a 4 x 6 factorial arrangement was used, with four initial pH values (5.5, 6.5, 7.5, and 8.5) and six sampling times (0, 4, 8, 12, 16 and 20 days). Every four days, three bottles from each treatment were removed and centrifuged at 9000 rpm for 20 min at 20 °C. The supernatant was filtered with 0.45 μm polypropylene filters and used to determine the pH, GA_3_, IAA and VFAs.

### Analytical methods

#### Manure characterization

The manure was dried at 103 °C, triturated, and sieved (openings 0.25 mm). The pH, total solids, and volatile solids were determined according to standard methods (APHA 2017).

#### Volatile fatty acids determination

The VFAs composition was determined by gas chromatography using an Agilent 7890A chromatograph with a capillary column HP-5 (film thickness, 0.25 μm; inside diameter, 0.32 mm; length, 30 m) and a flame ionization detector (FID). Nitrogen was used as the carrier gas at 3 mL/min, injector and detector temperatures 250 and 260 °C, respectively. An oven temperature program was initiated at 45 °C for 1.5 min and then increased by 60 °C/min until 250 °C at this temperature for 5 min. Acetic, propionic, butyric, isobutyric, isovaleric, and valeric acids from Sigma–Aldrich were used as analytical standards (Teniza-García et al. 2015).

#### IAA and GA_3_ determination

The pH of the supernatant was adjusted to 9 with NaOH (10 N) and extracted with an equal volume of ethyl acetate. The aqueous phase was recovered, the pH was adjusted to 1.5 with HCl (37%) and then extracted with an equal volume of ethyl acetate. The organic phase was evaporated at room temperature in a fume hood, and the residue was resuspended in a mixture of water–methanol (1:1) and acetic acid (0.2%), and filtered with a 0.22 μm nylon membrane (Ludwig-Müller and Cohen 2002). The quantification of IAA and GA_3_ was performed using a DAD-HPLC (Hewlett Packard) equipped with an Eclipse XDB-C18 column (4.6 mm ID × 250 mm × 5 μm) coupled to a Guard column Zorbax Reliance Cartridge (4.6 mm ID × 12.5 mm × 5 μm). The mobile phase consisted of water (A), methanol (B), and water–methanol (1:1, v/v) with 0.2% acetic acid (C). The elution program was as follows: 50% C, A from 50 to 25% (0 to 20 min), and B from 0 to 25% (0 to 20 min); then, from 20 to 27 min 50% C, 25% A and 25% B, flow rate 1 mL/min, injection volume 20 µL and column temperature 25 °C. IAA and GA_3_ were detected at 208 nm and 215 nm, respectively. To quantify IAA and GA_3_, calibration curves were developed using the corresponding IAA and GA_3_ analytical standards (Sigma–Aldrich).

#### Metagenomic analysis of anaerobic digestion processes

##### DNA extraction and amplicon sequencing

The digested samples at 0, 4, 8, and 20 days of each treatment were centrifuged at 9000 rpm for 20 min, and the cell biomass was dried at 60 °C for 5 h. The DNA was extracted from the mixture using the Power Soil DNA Isolation Kit (MO BIO Laboratories, Inc.). The quality of the DNA extracted was determined by the 260/280 spectrophotometric ratio, and the DNA concentration was adjusted to 50 ng/mL.

The library was prepared by amplification of the V4 region of the 16S rRNA gene using the PCR primers 515/806 and the HotStarTaq Plus Master Mix Kit (Qiagen, USA). The amplification program was as follows: one cycle of 94 °C for 3 min, 30 cycles of 94 °C for 30 s, 53 °C for 40 s, and 72 °C for 6 min. Library preparation and amplicon sequencing were performed at MR DNA Laboratory (Shallowater TX USA) using a PGM Ion Torrent sequencer.

##### Bioinformatic analysis

The sequencing data were analyzed on the Quantitative Insights Into Microbial Ecology (QIIME 2) (Bolyen et al. 2019) bioinformatic analysis platform and Cutadapt software. A total of 1,396,023 reads (raw) of approximately 300 bp were obtained, corresponding to the 13 sequenced samples.

Sequences greater than 300 bp and less than 295 bp were eliminated using Cutadapt software and then demultiplexed, and the primers were removed using QIIME 2. Then, sequences longer than 255 bp and shorter than 250 bp were eliminated. The remaining sequences were trimmed at the 5′ end, removing the first 20 bp using Cutadapt software. Subsequently, a quality filtering step was performed in QIIME 2 using a minimum quality score of 20 (Phred) and a tolerance of 3 consecutive bp with a quality of less than 20 before truncating a sequence. Finally, the chimeric sequences were eliminated, and the sequences with 100% similarity were grouped (formation of the ASVs) using the DADA2 algorithm included in QIIME 2.

The taxonomic assignation of the sequences of each sample was performed using a naive Bayes classifier trained on the SILVA 132 database (silva_132_99_16S) (Quast et al. 2013) for the 515F/806R primer pair.

### Statistical analysis

The results were analyzed with the factorial procedure ANOVA, and the differences between means were estimated using Tukey’s test at a significance level of 5%. Pearson correlation analysis of VFAs, IAA, and GA_3_ concentrations and the relative abundance of taxa at the genus level were conducted using RStudio (version 1.2.5033) software.

## Results

### Microbial community analysis

From among the expected products of the AD, VFAs were detected, but methane was not produced in any of the four AD, and thus only the hydrolytic and acidogenic processes were working in our experiments (Sträuber et al. 2012; Zhou et al. 2018). From the beginning of the AD, an incremental decrease in pH was observed, from 8.5 to 5.86, 7.5 to 5.58, 6.5 to 5.34, and 5.5 to 5.05, and after the 4th day, the pH values remained almost constant until the end of digestion (20 days), indicating the predominance of the acidogenic stage.

**Fig. 1:**
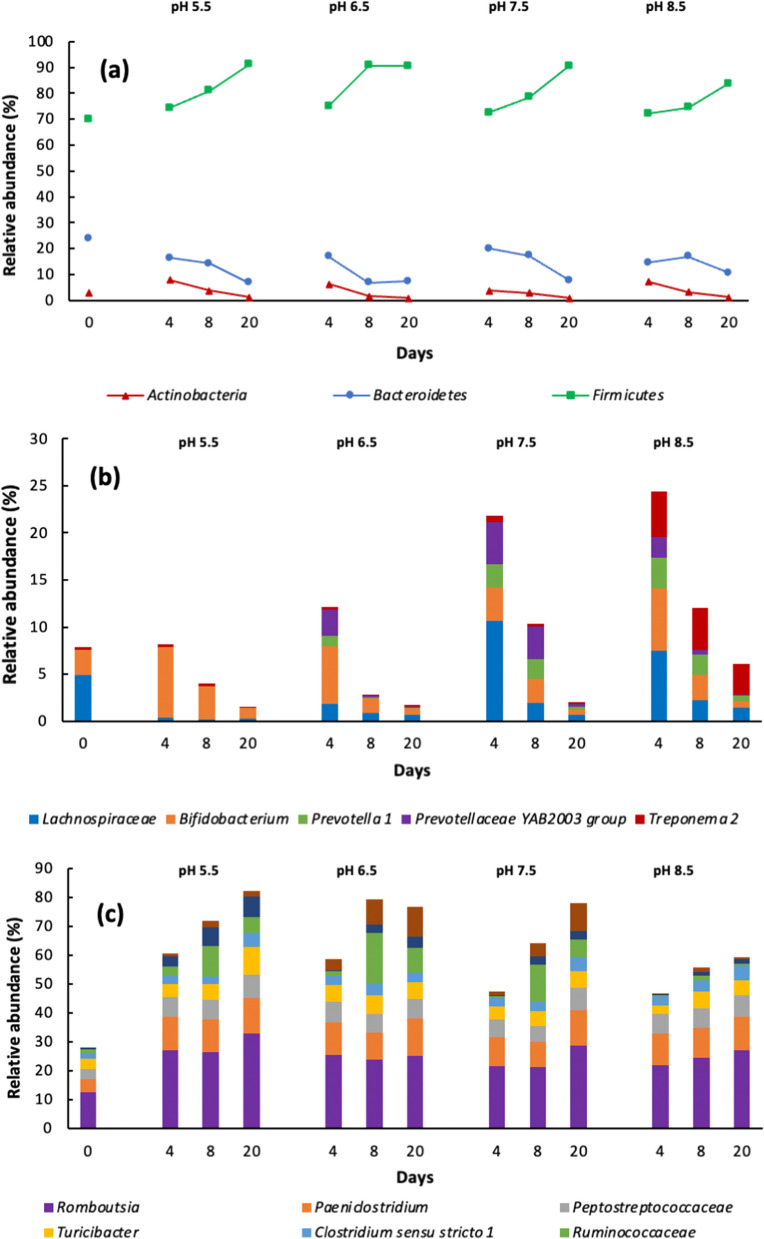
Dynamics of the relative abundance of the main bacterial taxa during the AD at initial pH 5.5, 6.5, 7.5 and 8.5. **a** phylum, **b** microorganisms with maximum growth from 0–4 days, and **c** microorganisms most abundant during the four digestions

The microbiome was dominated by the domain Bacteria (99.97%) with a small quantity of Archaea (0.03%). The taxa at the phylum level were *Firmicutes* (70–91%), *Bacteroidetes* (7–24%), and *Actinobacteria* (1–8%) (Fig. 1a), and these results are similar to those of other studies (Theuerl et al. 2020; Peng et al. 2018).

The population of *Firmicutes* increased continuously during the 20 days of the four digestions (Fig. 1a) and was the most abundant phylum. The pattern of this increase was similar among the four processes; *Firmicutes* include hydrolytic and acidogenic bacteria (Theuerl et al. 2020; Greses et al. 2020; Li et al. 2022; Khatami et al. 2021). *Bacteroidetes* are related to hydrolytic and acidogenic steps (Greses et al. 2020), but the conditions of the four AD tested were not favorable for their growth, and a diminution of the population throughout the entire AD process was observed. The abundance of *Actinobacteria* increased significantly during the first four days and then decreased appreciably until the end of digestion (Fig. 1a). *Actinobacteria* are hydrolytic bacteria and their abundance was related to the hydrolytic stage of the four AD.

During the first days of digestion, the hydrolytic stage was the main process of AD. In the four AD from 0–4 days, the most abundant genera were *Bifidobacterium* > *Treponema 2* > *Prevotellaceae YAB2003 group* > *Prevotella 1* > *Lachnospiraceae, Prevotella 1, Prevotellaceae YAB2003 group, Bifidobacterium*, *and Treponema 2*, which then diminished until the end of the fermentation (Fig. 1b). It could be considered that those microorganisms were mainly hydrolytic.

The most abundant genera present during the AD were *Romboutsia*, *Turicibacter, Peptostreptococcaceae, Clostridium sensu stricto 1, Paeniclostridium, Ruminococcaceae, Fonticella* and *Caproiciproducens*, which belong to the *Firmicutes* phylum (Fig. 1c, Fig. S1 and Table S1, supplementary material). The initial pH of the AD and the fermentation time influenced the microbial population: the relative abundance of *Romboutsia* and *Paeniclostridium* were the highest and they remained constant during the four digestions. *Clostridium *sensu stricto* 1* increased until 20 days at pH 5.5, 7.5 and 8.5, and its highest abundance was at pH 8.5. *Fonticella* was not detected at the beginning of fermentation; however, at pH 6.5 and 7.5 at 20 days, its abundance increased, whereas at pH 5.5 and 8.5 at 20 days, its abundance decreased appreciably. *Ruminococcaceae* was higher at 8 days and pH 6.5; by 20 days, its population had diminished approximately by 50%, whereas at pH 8.5, its population was negligible at 20 days. For *Caproiciproducens,* its abundance was negligible at the beginning of the fermentation but it increased with time, and its highest abundance was at pH 5.5, 20 days. The abundance of *Turicibacter* was increased at 20 days for all the AD studied, but at pH 5.5, it was the highest.

### Production of volatile fatty acids (VFAs) and their correlation with microbial communities

**Fig. 2:**
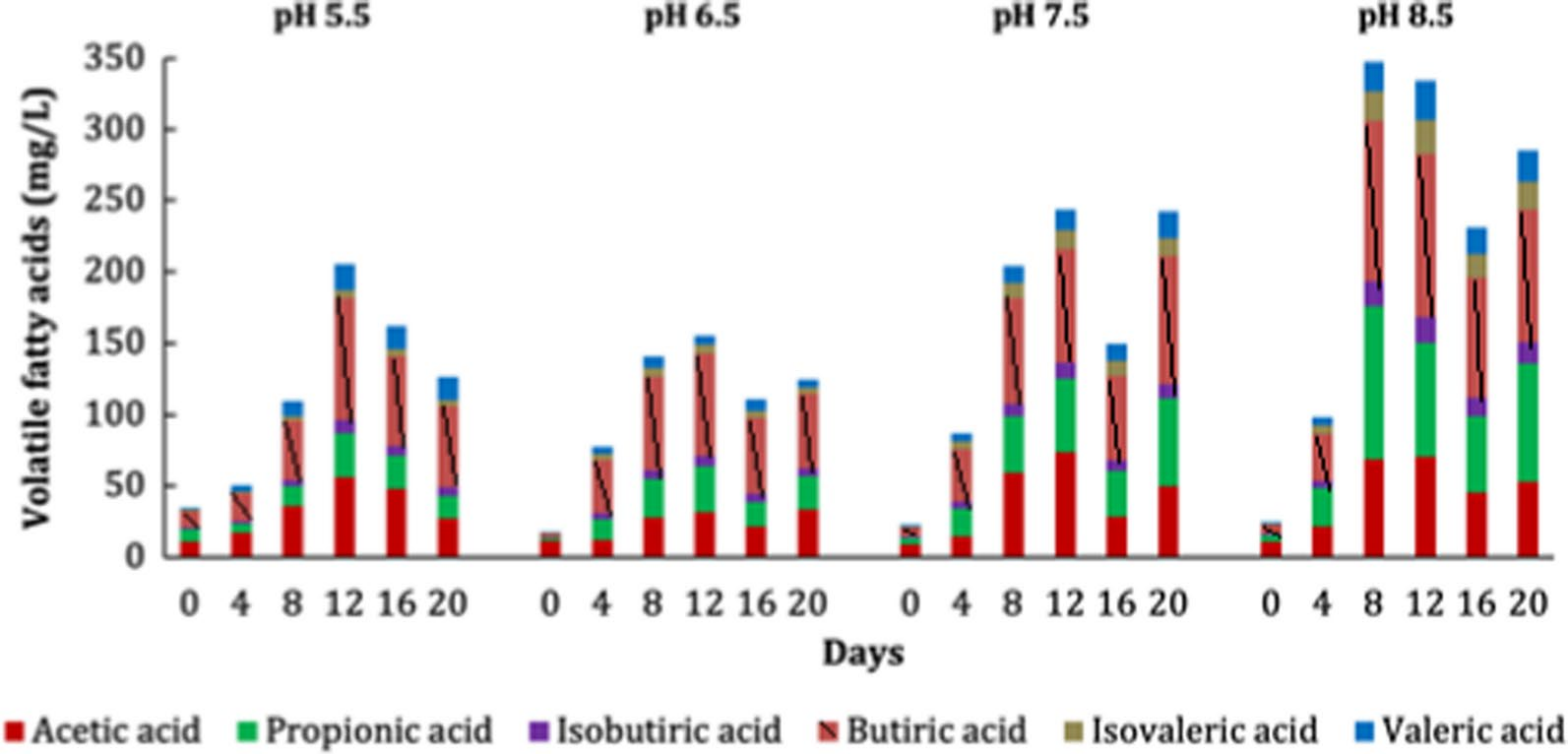
Production of VFAs during the anaerobic digestion of cow manure at initial pH values of 5.5, 6.5, 7.5, and 8.5

During the four digestions of cow manure (CM), the maximum production of VFAs was detected at 12 days (initial pH 5.5, 6.5, 7.5) and 8 days (initial pH 8.5), whereas the total production of AD was 653.3, 609.26, 836.5 and 1150.71 mg/L at pH 5.5, 6.5, 7.5, and 8.5, respectively (Fig. 2). The pH of the CM (7% total solids) was 6.5, but the previous treatment of the CM, acidic or alkaline, stimulated the production of VFAs. The VFAs production during acid fermentation, pH 5.5, was 7.3% higher than the production from untreated CM; however, with alkaline treatment, the production was substantially improved, 37% at pH 7.5 and 89% at pH 8.5.

Acetic (Hac), propionic (Hpro), butyric (Hbut), isobutyric (Hibut), valeric (Hval), and isovaleric acids (Hival) were produced in all four digestions (Fig. 2), and the greatest production was butyric > acetic > propionic . The highest production of each acid was different depending on the pH: 73.85 mg/L Hac (pH 7.5, 12 days), 106.73 mg/L Hpro (pH 8.5, 8 days), 114.53 mg/L Hbut, 17.73 mg/L Hibut, 24.4 mg/L Hival, and 27.55 mg/L Hval, all at pH 8.5, 12 days. These results agreed with Nzeteu et al. (2021) and Khatami et al. (2021). The proportions of the produced acids HBut:HAc:HPro:HVal:Hibut:Hival were also influenced by the initial pH, and the ratios were 41:27:15:5:9:3 (pH 5.5), 46:20:21:5:4:4 (pH 6.5), 35:33:14:5:6 (pH 7.5), and 37:23:17:6:7:8 (pH 8.5).

The possible relationships between the most abundant microbial communities and VFAs production in the four ADs were studied using Pearson correlations (Table S2, supplementary material). The production of Hac was highly correlated (> 0.8) with *Caproiciproducens* (at all pH values)*, Fonticella* (except pH 7.5), *Ruminococcaceae* (except pH 6.5 and 7.5), and *Turicibacter* (only at basic pH values). The correlation between Hpro, Hbut, Hibut, Hval and Hival was > 0.8 with *Turicibacter* (except with Hpro, pH 5.5); *Caproiciproducens* (except at pH 6.5); *Fonticella* (except Hpro and Hibut, pH 5.5); *Clostridium *sensu stricto 1 (except Hpro, pH 5.5); *Romboutsia* (except Hpro and Hibut, pH 5.5); *Peptostreptococcaceae* (except Hpro and Hibut, pH 5.5; Hpro and Hbut, pH 6.5, and all VFAs at pH 8.5); and *Paeniclostridium* only showed a correlation with VFAs at pH 7.5.

### Production of phytohormones and their correlation with microbial communities

During AD, the phytohormones GA_3_ and IAA were detected, and the amount was determined by the initial pH and the time of digestion. The highest and most continuous production of GA_3_ was observed at pH 6.5, and the highest concentration was 86.83 mg/L at 20 days (Table 1); at pH 5.5, the production was constant throughout the fermentation, but it was approximately 50% lower than that at pH 6.5. At initial pH values of 7.5 and 8.5, the maximum production of GA_3_ was reached at 4 days and 8 days, respectively, and then diminished until the end of the process (Castro-Ramos et al. 2021). The maximum production of IAA was detected at initial pH value of 5.5 and a small concentration of IAA at pH 6.5. The highest concentration of IAA was 4.9 mg/L at 4 days and pH 5.5 (Table 1), then a decrease was observed and again an increase at 20 days.

**Table 1:** Production of GA_3_ and IAA during the four anaerobic digestions

Time Day	GA_3_ (mg/L)				IAA (mg/L)			
	pH 5.5	pH 6.5	pH 7.5	pH 8.5	pH 5.5	pH 6.5	pH 7.5	pH 8.5
0	7.19	6.07	6.00	6.92	0.61	0.66	0.86	0.83
4	25.74	39.98	38.01	40.78	4.97	0.91	0.00	0.41
8	38.47	67.59	3.88	47.96	1.97	0.28	0.00	0.00
12	42.85	70.07	4.97	19.49	0.28	0.30	0.00	0.00
16	41.38	84.43	0.36	7.78	1.45	0.41	0.00	0.00
20	48.67	86.83	0.50	1.94	3.83	0.58	0.10	0.00

The anaerobic production of GA_3_ and IAA have been scarcely studied. Li et al. (2016) reported the production of GA_3_ in the anaerobic digestion, and detected GA_3_ during the AD of pig, dairy, and chicken manure, obtaining a maximum of 47.18 mg/L at 14 days from pig manure. The same authors (Li et al. 2016) found that IAA production started with a rapid increase at 8 days, and its highest production was 23.18, 23.41, and 13.30 mg/L with dairy, pig and chicken manure, respectively. Scaglia et al. (2017) reported that in AD of pig manure mixed with different organic wastes, after 55, 35, and 40 days, the IAA was 0.84, 3.24, and 3.23 mg/L, respectively. Scaglia et al. (2015) reported 9.94 mg/L of IAA in the neutral hydrophobic fraction of digested pig slurry.

**Fig. 3:**
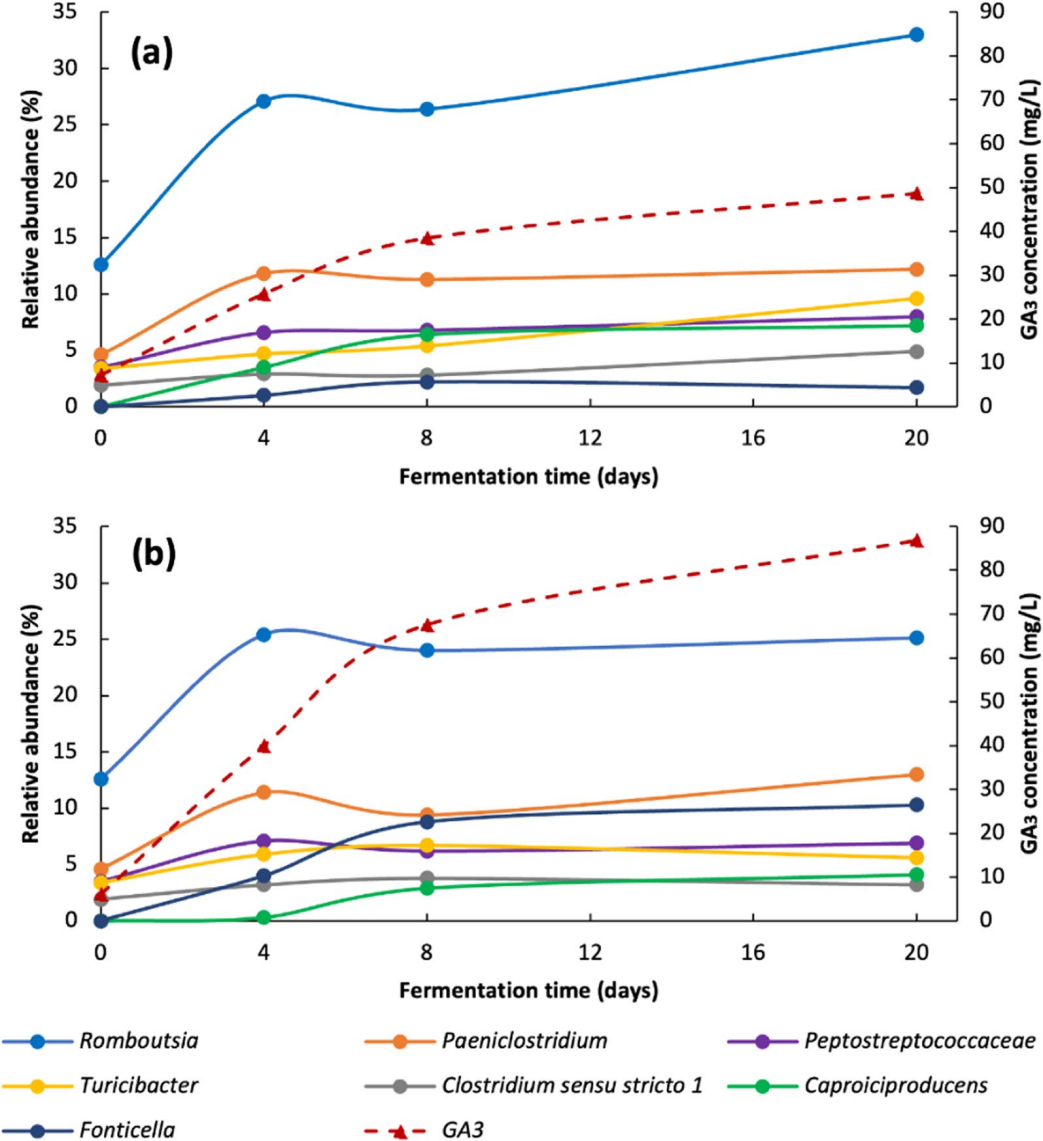
Relationship between the production pattern of GA_3_ and the microbial community abundance behavior at pH 5.5 (**a**) and 6.5 (**b**)

There was a correlation > 0.87 between the production of GA_3_ at pH 5.5 and 6.5 and the genera *Caproiciproducens*, *Clostridium *sensu stricto 1, *Romboutsia, Fonticella* and *Paeniclostridium*, in addition to the genera *Turicibacter* and *Peptostreptococcaceae* at pH 6.5 (Table S1, supplementary material). Figure 3 shows that the production pattern of GA_3_ and the relative abundance of the mentioned genera were very similar, and those genera are probably GA_3_ producers. In the case of IAA, there was not a strong correlation with any of the genera identified; to identify IAA producers, more studies need to be performed.

## Discussion

The AD of dairy cow manure with an initial pH 6.5 and adjusted to pH 5.5, 7.5, and 8.5, only involved the hydrolytic and acidogenic steps. Methane was not produced in any of the four AD due to the methanogenic bacteria are inhibited at pH lower than 6.6 (Teniza-García et al. 2015). Besides, the taxonomic assignment revealed that the relative abundance of archaea (methanogens) in all AD was lower than 0.33%, which was less than the relative archaea abundance of 4.2% necessary in an AD-producing methane process (Pore et al. 2016).

The pH had an important effect on the composition of the bacterial community of the four AD (Additional file 1: Table S1) (Huang et al. 2018). In this way, some of the genera detected in the cow manure diminished during the four AD, and some disappeared only at the end, whereas others were not detected initially, but their abundance increased during the AD (Additional file 1: Table S1). The pH also was very important for the surveillance of the microorganisms; at pH 5.5, the populations were the lowest in the four digestions, and *Prevotellaceae YAB2003 group* and *Prevotella 1* were not detected. At pH 6.5, the highest abundance was for *Bifidobacterium*; at pH 7.5, the highest abundance was for *Lachnospiraceae* and *Prevotellaceae YAB2003*; whereas the highest abundance was for *Prevotella 1* and *Treponema 2* at pH 8.5. *Lachnospiraceae* was able to efficiently degrade cellulose, hemicelluloses, lignin, and protein (Peng et al. 2018; Suksong et al. 2019). Duarte et al. (2017) found that *Prevotella* was dominant in the rumen microbiota, whereas *Prevotellaceae* produces cellulolytic enzymes, *Bifidobacterium* is a strong carbohydrate degrader (Nguyen et al. 2019; Pokusaeva et al. 2011), and *Treponema* is a hemicellulose degrader (Li et al. 2022).

The metabolic activity of the microbial community was different because different acidogenic pathways were strongly influenced by the initial pH (Jomnonkhaow et al. 2021). To maximize VFAs production, it is advisable to pretreat the cow manure to carry out AD at an initial pH of 8.5 or higher, which will maximize the economic advantages in the production of VFAs. Alkaline pretreatment constitutes a cheap and attractive alternative to maximize VFAs production. Several previous works also concluded that there was greater production of VFAs at alkaline pH (Huang et al. 2019, 2018; Bermúdez-Penabad et al. 2017; Khatami et al. 2021), probably because the hydrolysis of proteins and carbohydrates was favored, as well as the higher solubilization of the materials that occurs at basic pH compared to acidic pH (Lin and Li, 2018; Yuan et al. 2019). The anaerobic digestion of cow manure was shown to be an excellent alternative to the disposal of such material and the production of VFAs, products with a high economic relevance, was feasible.

All the microorganisms highly correlated with the production of VFAs were from the phylum *Firmicutes* (Additional file 1: Fig. S1), in agreement with Khatami et al. (2021). The genera *Peptostreptococcaceae*, *Clostridium, Caproiciproducens*, *Ruminococcaceae, Romboutsia, Clostridium sensu stricto,* and *Fonticella* have been reported to be VFAs producers (Wang et al. 2018, 2020; Khatami et al. 2021; Nzeteu et al. 2021; Li et al. 2022).

The GA_3_ and IAA amount was determined by the initial pH and the time of digestion. The highest production of GA_3_ during AD was reached at an initial pH of 6.5 for 16 days, while IAA production was favorable during AD at an initial pH of 5.5 for 4 days. There was a strong correlation between some genera from the phylum *Firmicutes* and VFAs and GA_3_ production, whereas no relationship was found with IAA production. To our knowledge, there are very few works where any of the bacterial genera studied in this work have been related to the production of GA_3_ and/or IAA. Dai et al. (2021) mentioned that IAA was positively correlated with *Romboutsia, Blautia, Bifidobacterium*, and *Ruminococcus torques group*. *Peptostreptococcaceae* (Younge et al. 2019) and *Turicibacter* (Yu et al. 2019) were reported to be correlated with IAA production.

In conclusion, anaerobic digestion of cow manure is an alternative to produce valuable products, as volatile fatty acids, gibberellic acid, and indole-3-acetic acid. The initial pH of the anaerobic digestion and the fermentation time had an important and different effect in the production of these products. The maximum amount of volatile fatty acids was achieved at an initial pH of 8.5, the main volatile fatty acid produced in all digestions was butyric acid, followed by acetic and propionic acids. For the highest production of gibberellic acid, it is recommended to carry out the anaerobic digestion at an initial pH of 6.5 for 16 days; while to produce the highest quantity of indole-3-acetic acid, it is suitable to perform the anaerobic digestion at an initial pH of 5.5 for 4 days.

## Supplementary Information

**Additional file 1: Table S1.** Microorganism relative abundance of the cow manure anaerobic digestion. **Table S2.** Pearson correlations between genera and volatile fatty acids, IAA and GA_3_ at initial pH values of 5.5, 6.5, 7.5, and 8.5. **Figure S1.** Phylogenetic tree of the most abundant genera found in the four digestions

## Data Availability

Ion PGM sequencing data is in the NCBI Sequence Read Archive database SUB11548967, Bioproject PRJNA844385.
