# Supplementary material for: Effect of the initial pH on the anaerobic digestion process of dairy cattle manure
Source: AMB Express. 2022 Dec 28;12:162. doi: 10.1186/s13568-022-01486-8 (PMC9797631; doi:10.1186/s13568-022-01486-8)
Supplement: Supplementary file 1 — Additional file 1: Table S1. Microorganism relative abundance of the cow manure anaerobic digestion. Table S2. Pearson correlations between genera and volatile fatty acids, IAA and GA3 at initial pH values of 5.5, 6.5, 7.5, and 8.5. Figure S1. Phylogenetic tree of the most abundant genera found in the four digestions [file 13568_2022_1486_MOESM1_ESM.docx]

**Additional material**

**Table S1.** Microorganism relative abundance of the cow manure anaerobic digestion

|  | **Taxon** | **0 d**  **(%)** | **4 d,**  **pH 5.5**  **(%)** | **8 d,**  **pH 5.5**  **(%)** | **20 d,**  **pH 5.5**  **(%)** | **4 d,**  **pH 6.5**  **(%)** | **8 d,**  **pH 6.5**  **(%)** | **20 d,**  **pH 6.5**  **(%)** | **4 d,**  **pH 7.5**  **(%)** | **8 d,**  **pH 7.5 (%)** | **20 d,**  **pH 7.5 (%)** | **4 d,**  **pH 8.5 (%)** | **8 d,**  **pH 8.5 (%)** | **20 d,**  **pH 8.5 (%)** |
| --- | --- | --- | --- | --- | --- | --- | --- | --- | --- | --- | --- | --- | --- | --- |
| **Domain** | *Bacteria* | 99.97 | 100 | 100 | 100 | 99.9 | 99.9 | 99.9 | 99.7 | 99.8 | 99.9 | 99.7 | 99.9 | 100 |
|  | *Archaea* | 0.03 | 0 | 0 | 0 | 0.07 | 0.03 | 0.10 | 0.32 | 0.14 | 0.08 | 0.31 | 0.10 | 0.0 |
| **Phylum** | *Firmicutes* | 69.6 | 74.3 | 81.0 | 91.1 | 75.1 | 90.7 | 90.6 | 72.6 | 78.4 | 90.6 | 72.2 | 74.5 | 83.7 |
|  | *Bacteroidetes* | 23.9 | 16.5 | 14.2 | 6.8 | 17.0 | 6.8 | 7.5 | 21.6 | 17.3 | 7.8 | 14.6 | 16.9 | 10.6 |
|  | *Actinobacteria* | 3.0 | 8.0 | 3.9 | 1.4 | 6.3 | 1.8 | 1.0 | 3.9 | 3.0 | 1.0 | 7.2 | 3.3 | 1.5 |
|  | *Tenericutes* | 2.2 | 0.4 | 0.3 | 0.1 | 0.3 | 0.1 | 0.1 | 0.3 | 0.2 | 0.1 | 0.4 | 0.3 | 0.3 |
|  | *Spirochaetes* | 0.3 | 0.3 | 0.3 | 0.1 | 0.3 | 0.1 | 0.2 | 0.7 | 0.3 | 0.2 | 4.8 | 4.4 | 3.3 |
| **Class** | *Clostridia* | 63.4 | 65.2 | 74.0 | 79.5 | 67.3 | 82.5 | 83.7 | 65.7 | 71.5 | 83.2 | 67.7 | 67.0 | 76.9 |
|  | *Bacteroidia* | 23.9 | 16.5 | 14.2 | 6.8 | 17.0 | 6.8 | 7.5 | 21.6 | 17.3 | 7.8 | 14.6 | 16.9 | 10.6 |
|  | *Erysipelotrichia* | 4.7 | 6.6 | 5.6 | 9.8 | 6.7 | 7.0 | 5.9 | 5.7 | 5.6 | 6.1 | 3.5 | 6.1 | 5.5 |
|  | *Actinobacteria* | 2.7 | 7.5 | 3.5 | 1.2 | 6.1 | 1.6 | 0.7 | 3.5 | 2.5 | 0.6 | 6.6 | 2.6 | 0.7 |
|  | *Mollicutes* | 2.2 | 0.4 | 0.3 | 0.1 | 0.3 | 0.1 | 0.1 | 0.3 | 0.2 | 0.1 | 0.4 | 0.3 | 0.3 |
|  | *Bacilli* | 0.7 | 1.4 | 1.0 | 1.5 | 0.8 | 0.8 | 0.8 | 0.7 | 0.7 | 1.0 | 0.6 | 0.9 | 1.0 |
|  | *Spirochaetia* | 0.3 | 0.3 | 0.3 | 0.1 | 0.3 | 0.1 | 0.2 | 0.7 | 0.3 | 0.2 | 4.8 | 4.4 | 3.3 |
| **Order** | *Clostridiales* | 63.4 | 65.2 | 74.0 | 79.5 | 67.3 | 82.5 | 83.7 | 65.6 | 71.5 | 83.2 | 67.7 | 67.0 | 76.9 |
|  | *Bacteroidales* | 23.9 | 16.5 | 14.2 | 6.8 | 17.0 | 6.8 | 7.5 | 21.6 | 17.3 | 7.8 | 14.6 | 16.9 | 10.6 |
|  | *Erysipelotrichales* | 4.7 | 6.6 | 5.6 | 9.8 | 6.7 | 7.0 | 5.9 | 5.7 | 5.6 | 6.1 | 3.5 | 6.1 | 5.5 |
|  | *Bifidobacteriales* | 2.7 | 7.5 | 3.5 | 1.2 | 6.1 | 1.6 | 0.7 | 3.5 | 2.5 | 0.6 | 6.6 | 2.6 | 0.7 |
|  | *Mollicutes* | 2.2 | 0.3 | 0.2 | 0.1 | 0.3 | 0.1 | 0.1 | 0.3 | 0.2 | 0.1 | 0.4 | 0.3 | 0.3 |
|  | *Selenomonadales* | 0.8 | 1.0 | 0.5 | 0.3 | 0.3 | 0.3 | 0.2 | 0.6 | 0.5 | 0.2 | 0.4 | 0.5 | 0.3 |
|  | *Bacillales* | 0.4 | 0.6 | 0.7 | 1.2 | 0.6 | 0.8 | 0.6 | 0.5 | 0.7 | 0.8 | 0.4 | 0.9 | 0.9 |
|  | *Spirochaetales* | 0.3 | 0.3 | 0.3 | 0.1 | 0.3 | 0.1 | 0.2 | 0.7 | 0.3 | 0.2 | 4.8 | 4.4 | 3.3 |
| **Family** | *Ruminococcaceae* | 22.9 | 9.0 | 19.2 | 14.1 | 4.3 | 23.0 | 16.3 | 2.4 | 18.2 | 10.9 | 2.4 | 5.6 | 9.1 |
|  | *Peptostreptococcaceae* | 21.0 | 46.1 | 45.1 | 53.9 | 45.1 | 40.5 | 46.0 | 38.3 | 35.9 | 49.5 | 40.5 | 42.3 | 47.0 |
|  | *Bacteroidaceae* | 10.1 | 8.1 | 4.9 | 2.7 | 6.2 | 2.4 | 3.1 | 7.9 | 4.9 | 3.0 | 4.5 | 5.4 | 3.5 |
|  | *Rikenellaceae* | 9.2 | 6.9 | 7.4 | 3.3 | 6.1 | 3.6 | 3.3 | 5.7 | 5.7 | 3.0 | 3.6 | 6.2 | 4.5 |
|  | *Lachnospiraceae* | 8.2 | 2.8 | 2.1 | 2.3 | 5.4 | 3.1 | 3.0 | 15.3 | 5.4 | 3.7 | 12.9 | 8.0 | 7.4 |
|  | *Erysipelotrichaceae* | 4.7 | 6.6 | 5.6 | 9.8 | 6.7 | 7.0 | 5.9 | 5.7 | 5.6 | 6.1 | 3.5 | 6.1 | 5.5 |
|  | *Bifidobacteriaceae* | 2.7 | 7.5 | 3.5 | 1.2 | 6.1 | 1.6 | 0.7 | 3.5 | 2.5 | 0.6 | 6.6 | 2.6 | 0.7 |
|  | *Prevotellaceae* | 2.2 | 0.2 | 0.3 | 0.1 | 4.0 | 0.4 | 0.4 | 7.1 | 5.9 | 1.2 | 5.6 | 3.6 | 1.3 |
|  | *Clostridiaceae* | 1.9 | 4.9 | 5.9 | 7.7 | 9.1 | 14.2 | 14.9 | 6.0 | 9.0 | 16.2 | 6.1 | 6.5 | 7.5 |
|  | *Christensenellaceae* | 1.9 | 0.2 | 0.1 | 0.0 | 0.3 | 0.1 | 0.1 | 0.6 | 0.3 | 0.2 | 0.8 | 0.9 | 0.8 |
|  | *Spirochaetaceae* | 0.3 | 0.3 | 0.3 | 0.1 | 0.3 | 0.1 | 0.2 | 0.7 | 0.3 | 0.2 | 4.8 | 4.4 | 3.3 |
| **Genus** | *Ruminococcaceae UCG-005* | 13.3 | 0.7 | 0.8 | 0.5 | 0.4 | 0.2 | 0.3 | 0.2 | 0.2 | 0.1 | 0.3 | 0.6 | 1.0 |
|  | *Romboutsia* | 12.6 | 27.1 | 26.4 | 33.0 | 25.4 | 24.0 | 25.1 | 21.6 | 21.3 | 28.8 | 22.0 | 24.4 | 27.1 |
|  | *Bacteroides* | 10.1 | 8.1 | 4.9 | 2.7 | 6.2 | 2.4 | 3.1 | 7.9 | 4.9 | 3.0 | 4.5 | 5.4 | 3.5 |
|  | *Rikenellaceae RC9 gut group* | 6.6 | 5.5 | 6.2 | 2.5 | 5.3 | 3.1 | 2.5 | 4.7 | 4.7 | 2.5 | 3.0 | 5.4 | 4.0 |
|  | *Lachnospiraceae;* | 4.9 | 0.4 | 0.2 | 0.3 | 1.9 | 0.9 | 0.7 | 10.7 | 2.0 | 0.7 | 7.5 | 2.3 | 1.5 |
|  | *Paeniclostridium* | 4.6 | 11.8 | 11.3 | 12.2 | 11.4 | 9.4 | 13.0 | 10.2 | 8.6 | 12.3 | 10.9 | 10.6 | 11.5 |
|  | *Peptostreptococcaceae;* | 3.5 | 6.6 | 6.8 | 8.0 | 7.1 | 6.2 | 6.9 | 5.9 | 5.5 | 7.6 | 6.9 | 6.6 | 7.5 |
|  | *Turicibacter* | 3.4 | 4.7 | 5.4 | 9.6 | 5.9 | 6.7 | 5.6 | 4.5 | 5.2 | 5.9 | 2.7 | 5.7 | 5.1 |
|  | *Clostridiales;* | 3.1 | 0.5 | 0.5 | 0.4 | 0.5 | 0.5 | 1.9 | 0.7 | 0.4 | 0.9 | 0.8 | 0.6 | 0.9 |
|  | *Bifidobacterium* | 2.7 | 7.5 | 3.5 | 1.2 | 6.1 | 1.6 | 0.7 | 3.5 | 2.5 | 0.6 | 6.6 | 2.6 | 0.7 |
|  | *Clostridiales Family XIII AD3011* | 2.3 | 1.1 | 0.6 | 0.6 | 1.6 | 0.7 | 0.8 | 1.3 | 1.3 | 1.1 | 2.1 | 1.8 | 2.0 |
|  | *Eubacterium coprostanoligenes* | 2.1 | 0.2 | 0.2 | 0.1 | 0.3 | 0.2 | 0.1 | 0.1 | 0.3 | 0.2 | 0.3 | 0.4 | 3.8 |
|  | *Clostridium sensu stricto 1* | 1.9 | 2.9 | 2.8 | 4.9 | 3.2 | 3.8 | 3.2 | 3.0 | 3.3 | 4.8 | 3.4 | 4.1 | 5.4 |
|  | *Ruminococcaceae UCG-014* | 1.9 | 0.3 | 0.1 | 0.1 | 0.3 | 0.1 | 0.1 | 0.2 | 0.2 | 0.1 | 0.2 | 0.3 | 0.3 |
|  | *Alistipes* | 1.8 | 0.5 | 0.6 | 0.4 | 0.2 | 0.2 | 0.4 | 0.2 | 0.5 | 0.3 | 0.3 | 0.4 | 0.3 |
|  | *Mollicutes RF39;* | 1.8 | 0.2 | 0.2 | 0.1 | 0.2 | 0.1 | 0.1 | 0.3 | 0.2 | 0.1 | 0.4 | 0.2 | 0.2 |
|  | *Christensenellaceae R-7 group* | 1.8 | 0.2 | 0.1 | 0.0 | 0.3 | 0.1 | 0.1 | 0.6 | 0.3 | 0.2 | 0.8 | 0.8 | 0.8 |
|  | *Ruminococcaceae;* | 1.6 | 3.1 | 10.6 | 5.6 | 1.6 | 17.6 | 8.7 | 0.5 | 12.8 | 6.1 | 0.3 | 1.6 | 0.6 |
|  | *Prevotellaceae UCG-004* | 1.0 | 0.1 | 0.1 | 0.0 | 0.0 | 0.0 | 0.0 | 0.1 | 0.2 | 0.6 | 0.0 | 0.8 | 0.6 |
|  | *Prevotellaceae UCG-003* | 0.9 | 0.1 | 0.2 | 0.1 | 0.1 | 0.1 | 0.2 | 0.1 | 0.1 | 0.1 | 0.0 | 0.1 | 0.0 |
|  | *Lachnospiraceae NK3A20 group* | 0.8 | 0.6 | 0.4 | 0.2 | 1.0 | 0.4 | 0.5 | 1.2 | 0.8 | 0.7 | 1.3 | 1.4 | 1.0 |
|  | *Cellulosilyticum* | 0.7 | 0.7 | 0.8 | 1.2 | 1.4 | 1.2 | 1.1 | 1.6 | 1.3 | 1.5 | 0.9 | 1.2 | 1.5 |
|  | *Rikenellaceae dgA-11 gut group* | 0.7 | 1.0 | 0.6 | 0.4 | 0.6 | 0.2 | 0.4 | 0.8 | 0.5 | 0.3 | 0.4 | 0.4 | 0.2 |
|  | *Terrisporobacter* | 0.3 | 0.6 | 0.6 | 0.7 | 1.3 | 0.9 | 1.0 | 0.7 | 0.6 | 0.7 | 0.8 | 0.7 | 0.9 |
|  | *Ruminiclostridium 1* | 0.3 | 0.0 | 0.0 | 0.0 | 0.1 | 1.2 | 1.7 | 0.0 | 0.1 | 0.6 | 0.0 | 0.3 | 0.5 |
|  | *Caproiciproducens* | 0.01 | 3.5 | 6.4 | 7.2 | 0.3 | 2.9 | 4.1 | 0.5 | 3.0 | 3.1 | 0.4 | 1.3 | 1.5 |
|  | *Fonticella* | 0.0 | 1.0 | 2.2 | 1.7 | 4.0 | 8.8 | 10.3 | 1.3 | 4.4 | 9.6 | 0.2 | 1.4 | 0.8 |
|  | *Ruminiclostridium* | 0.0 | 0.9 | 1.0 | 0.4 | 0.8 | 0.5 | 1.0 | 0.0 | 0.7 | 0.4 | 0.0 | 0.0 | 0.2 |
|  | *Prevotella 1* | 0.0 | 0.0 | 0.0 | 0.0 | 1.1 | 0.1 | 0.1 | 2.5 | 2.1 | 0.3 | 3.3 | 2.2 | 0.6 |
|  | *Prevotellaceae YAB2003 group* | 0.0 | 0.0 | 0.0 | 0.0 | 2.7 | 0.2 | 0.1 | 4.4 | 3.5 | 0.3 | 2.2 | 0.5 | 0.1 |
|  | *Clostridium sensu stricto 13* | 0.0 | 0.1 | 0.1 | 0.1 | 0.5 | 0.1 | 0.1 | 1.0 | 0.5 | 0.0 | 1.3 | 0.3 | 0.3 |
|  | *Mogibacterium* | 0.4 | 0.4 | 0.2 | 0.3 | 0.7 | 0.2 | 0.2 | 0.5 | 0.6 | 0.4 | 0.8 | 0.8 | 1.2 |
|  | *Treponema 2* | 0.3 | 0.3 | 0.3 | 0.1 | 0.3 | 0.1 | 0.2 | 0.7 | 0.3 | 0.2 | 4.8 | 4.4 | 3.2 |
|  | *Lachnospiraceae FCS020 group* | 0.0 | 0.0 | 0.0 | 0.0 | 0.0 | 0.0 | 0.0 | 0.0 | 0.0 | 0.0 | 0.0 | 0.5 | 1.5 |
|  | *Sharpea* | 0.2 | 1.5 | 0.0 | 0.0 | 0.4 | 0.2 | 0.0 | 0.8 | 0.2 | 0.1 | 0.2 | 0.1 | 0.0 |

Table S2. Pearson correlations between genera and volatile fatty acids, IAA and GA_3_ at initial pH values of 5.5, 6.5, 7.5, and 8.5

|  | pH 5.5 | | | | | | | |
| --- | --- | --- | --- | --- | --- | --- | --- | --- |
|  | IAA | GA | Hac | Hpro | Hibut | Hbut | Hival | Hval |
| *Caproiciproducens* | 0.47 | **0.99** | **0.89** | **0.80** | **0.90** | **0.94** | **0.95** | **0.94** |
| *Turicibacter* | 0.46 | **0.88** | 0.53 | 0.79 | **0.92** | **0.92** | **0.92** | **0.93** |
| *Peptostreptococcaceae* | 0.73 | **0.96** | 0.72 | 0.60 | 0.79 | **0.85** | **0.86** | **0.85** |
| *Clostridium* *sensu stricto 1* | 0.58 | **0.87** | 0.47 | 0.69 | **0.86** | **0.88** | **0.88** | **0.88** |
| *Romboutsia* | 0.77 | **0.94** | 0.67 | 0.56 | 0.76 | **0.82** | **0.83** | **0.82** |
| *Fonticella* | 0.34 | **0.90** | **0.97** | 0.73 | 0.78 | **0.83** | **0.84** | **0.83** |
| *Paeniclostridium* | **0.81** | **0.87** | 0.68 | 0.41 | 0.62 | 0.70 | 0.71 | 0.70 |
| *Ruminococcaceae* | -0.03 | 0.68 | **0.97** | 0.70 | 0.63 | 0.66 | 0.67 | 0.66 |
|  | pH 6.5 | | | | | | | |
|  | IAA | GA | Hac | Hpro | Hibut | Hbut | Hival | Hval |
| *Caproiciproducens* | -0.57 | **0.94** | **1.00** | **0.86** | 0.75 | 0.78 | 0.62 | 0.65 |
| *Turicibacter* | -0.26 | 0.76 | 0.56 | **0.90** | **0.97** | **0.95** | **1.00** | **0.99** |
| *Peptostreptococcaceae* | 0.18 | 0.77 | 0.48 | 0.76 | **0.84** | 0.79 | **0.82** | 0.79 |
| *Clostridium sensu stricto 1* | -0.34 | **0.80** | 0.63 | **0.94** | **0.98** | **0.97** | **1.00** | **1.00** |
| *Romboutsia* | 0.05 | **0.82** | 0.55 | **0.84** | **0.90** | **0.86** | **0.89** | **0.87** |
| *Fonticella* | -0.46 | **0.99** | **0.95** | **0.97** | **0.91** | **0.92** | **0.82** | **0.83** |
| *Paeniclostridium* | 0.18 | **0.84** | 0.60 | 0.75 | 0.78 | 0.74 | 0.72 | 0.69 |
| *Ruminococcaceae* | -0.88 | 0.68 | 0.77 | **0.83** | 0.77 | **0.83** | 0.75 | 0.79 |
|  | pH 7.5 | | | | | | | |
|  | IAA | GA | Hac | Hpro | Hibut | Hbut | Hival | Hval |
| *Caproiciproducens* | -0.60 | -0.56 | **0.98** | **0.93** | **0.95** | **0.97** | **0.96** | **0.93** |
| *Turicibacter* | -0.76 | -0.27 | **0.85** | **0.98** | **0.99** | **0.99** | **0.99** | **0.97** |
| *Peptostreptococcaceae* | -0.74 | -0.02 | 0.60 | **0.90** | **0.88** | **0.87** | **0.87** | **0.89** |
| *Clostridium sensu stricto 1* | -0.64 | -0.27 | 0.72 | **0.97** | **0.92** | **0.93** | **0.92** | **0.97** |
| *Romboutsia* | -0.76 | -0.08 | 0.67 | **0.92** | **0.91** | **0.90** | **0.91** | **0.92** |
| *Fonticella* | -0.47 | -0.51 | 0.78 | **0.98** | **0.90** | **0.92** | **0.90** | **0.99** |
| *Paeniclostridium* | -0.81 | 0.13 | 0.53 | **0.82** | **0.83** | **0.81** | **0.82** | **0.82** |
| *Ruminococcaceae* | -0.44 | -0.59 | **0.92** | 0.59 | 0.69 | 0.70 | 0.70 | 0.58 |
|  | pH 8.5 | | | | | | | |
|  | IAA | GA | Hac | Hpro | Hibut | Hbut | Hival | Hval |
| *Caproiciproducens* | -0.99 | 0.07 | **0.94** | **0.95** | **0.97** | **0.96** | **0.99** | **1.00** |
| *Turicibacter* | -0.87 | 0.05 | **0.93** | **0.92** | **0.92** | **0.91** | **0.89** | **0.90** |
| *Peptostreptococcaceae* | -0.78 | 0.31 | 0.65 | 0.68 | 0.70 | 0.71 | 0.76 | 0.74 |
| *Clostridium sensu stricto 1* | -0.91 | -0.02 | 0.78 | **0.81** | **0.85** | **0.83** | **0.90** | **0.91** |
| *Romboutsia* | -0.91 | 0.24 | **0.80** | **0.83** | **0.85** | **0.85** | **0.89** | **0.88** |
| *Fonticella* | -0.91 | 0.41 | **0.99** | **0.98** | **0.96** | **0.97** | **0.93** | **0.91** |
| *Paeniclostridium* | -0.78 | 0.40 | 0.67 | 0.70 | 0.71 | 0.72 | 0.76 | 0.73 |
| *Ruminococcaceae* | 0.09 | 0.02 | 0.14 | 0.09 | 0.05 | 0.06 | -0.05 | -0.04 |

Figure S1

Figure S1. Phylogenetic tree of the most abundant genera found in the four digestions
